# Complete Genome Sequence of *Campylobacter iguaniorum* Strain 1485E^T^, Isolated from a Bearded Dragon (*Pogona vitticeps*)

**DOI:** 10.1128/genomeA.00844-14

**Published:** 2014-08-21

**Authors:** Maarten J. Gilbert, William G. Miller, Emma Yee, Marja Kik, Jaap A. Wagenaar, Birgitta Duim

**Affiliations:** a Department of Infectious Diseases and Immunology, Faculty of Veterinary Medicine, Utrecht University, Utrecht, The Netherlands; b Produce Safety and Microbiology Research Unit, Agricultural Research Service, U.S. Department of Agriculture, Albany, California, USA; c Department of Pathobiology, Faculty of Veterinary Medicine, Utrecht University, Utrecht, The Netherlands; d Central Veterinary Institute of Wageningen UR, Lelystad, The Netherlands; e WHO Collaborating Center for Campylobacter/OIE Reference Laboratory for Campylobacteriosis, Utrecht, The Netherlands

## Abstract

*Campylobacter iguaniorum* has been isolated from reptiles. This *Campylobacter* species is genetically related to *Campylobacter fetus* and *Campylobacter hyointestinalis*. Here we present the first whole-genome sequence for this species.

## GENOME ANNOUNCEMENT

*Campylobacter iguaniorum* is genetically related to, but distinct from, the species *Campylobacter fetus* and *Campylobacter hyointestinalis* and has recently been proposed as a novel *Campylobacter* species (Gilbert et al., submitted for publication). Reptiles, chelonians, and lizards in particular appear to be the primary reservoir of this *Campylobacter* species (1). The pathogenicity of this species is unknown; strains have been recovered from reptiles with and without clinical signs of disease (1, 2). Here, we report the first whole-genome sequence of the *C*. *iguaniorum* type strain 1485E^T^ (LMG 28143^T^), isolated from a bearded dragon (*Pogona vitticeps*).

Sequencing was performed using shotgun and paired-end reads obtained on a Roche 454 FLX genome sequencer. Using the Newbler assembler (v. 2.6), we assembled a total of 220,914 454 reads into a single chromosomal scaffold of 12 contigs and a single megaplasmid contig, providing a draft genome sequence with a coverage of 68×. All 454 base calls were validated using 1,570,644 Illumina MiSeq reads, providing an additional 178× coverage. Scaffold gaps were filled as described (3). Sequences across the contig junctions were confirmed with Sanger sequencing. Homopolymeric GC tracts were characterized using the high-depth MiSeq reads.

The circular genome size of *C*. *iguaniorum* strain 1485E^T^ is 1,684,608 bp, with an average G+C content of 35.9%. A 70,030-bp megaplasmid is present. Protein-, rRNA-, and tRNA-encoding genes were identified as described (3). The genome was annotated based on *C. fetus* subsp. *testudinum* strain 03-427^T^ (accession number CP006833) (4, 5), with further annotation using Artemis (6), the identification of Pfam domains [v.26.0 (7)], and BLASTP comparisons to proteins in the NCBI nonredundant (nr) database. The chromosomal genome encodes 1663 putative protein-coding genes (including 18 probable pseudogenes), 43 tRNA genes, and 3 rRNA operons. The megaplasmid encodes an additional 111 putative protein-coding genes. A total of 34 homopolymeric GC tracts (≥8 bp) were identified, of which 23 were hypervariable. Many of these hypervariable GC tracts reside in surface-structure-related genes, as in other campylobacters; notably, however, eight are located within autotransporter domain-containing genes, whose role in *C. iguaniorum* biology remains to be determined.

In contrast to *C. fetus*, strain 1485E^T^ does not encode an S-layer. Also, a defined lipooligosaccharide (LOS) region, bounded by *waa* genes and containing multiple glycosyltransferases, is absent from strain 1485E^T^; instead, five separate predicted glycosylation regions were identified. One of these regions is >50 kb and has a deviant G+C content (30.0%), suggesting possible acquisition via lateral transfer. A clustered regularly interspaced short palindromic repeats (CRISPR)*-*Cas system is present. Multiple genes encoding respiratory enzymes not identified to date within the *C. fetus* group are also present. Interestingly, as in reptile-associated *C. fetus* subsp. *testudinum*, a putative tricarballylate catabolism pathway was identified.

The whole-genome sequence of strain 1485E^T^ supports the proposal of *C*. *iguaniorum*. Further genome analysis and comparison can provide valuable insights into host adaptation, virulence, taxonomic structure, and evolution of this novel reptile-associated *Campylobacter* species.

### Nucleotide sequence accession numbers.

The complete genome sequence of *C*. *iguaniorum* strain 1485E^T^ has been deposited in GenBank under accession numbers CP009043 and CP009044.

## References

[1] Gilbert MJ Kik M Timmerman AJ Severs TT Kusters JG Duim B Wagenaar JA 2014 Occurrence, diversity, and host association of intestinal *Campylobacter*, *Arcobacter*, and *Helicobacter* in reptiles. PLoS One 9:e101599. 10.1371/journal.pone.0101599 24988130 PMC4079654

[2] Benejat L Gravet A Sifré E Ben Amor S Quintard B Mégraud F Lehours P 2014 Characterization of a *Campylobacter fetus*-like strain isolated from the faeces of a sick leopard tortoise (*Stigmochelys pardalis*) using matrix-assisted laser desorption/ionization time of flight as an alternative to bacterial 16S rDNA phylogeny. Lett. Appl. Microbiol. 58:338–343. 10.1111/lam.12194 24313345

[3] Merga JY Winstanley C Williams NJ Yee E Miller WG 2013 Complete genome sequence of the *Arcobacter butzleri* cattle isolate 7h1h. Genome Announc. 1(4):e00655-13. 10.1128/genomeA.00655-13 23969057 PMC3751612

[4] Gilbert MJ Miller WG Yee E Blaser MJ Wagenaar JA Duim B 2013 Complete genome sequence of *Campylobacter fetus* subsp. *testudinum* strain 03–427^T^. Genome Announc. 1(6):e01002-13. 10.1128/genomeA.01002-13 24336365 PMC3861418

[5] Fitzgerald C Tu ZC Patrick M Stiles T Lawson AJ Santovenia M Gilbert MJ van Bergen M Joyce K Pruckler J Stroika S Duim B Miller WG Loparev VL Sinnige JC Fields PI Tauxe RV Blaser MJ Wagenaar JA 4 6 2014 Description of *Campylobacter fetus* subsp. *testudinum* subsp. nov., isolated from humans and reptiles. Int. J. Syst. Evol. Microbiol. 10.1099/ijs.0.057778-0 24899653

[6] Rutherford K Parkhill J Crook J Horsnell T Rice P Rajandream MA Barrell B 2000 Artemis: sequence visualization and annotation. Bioinformatics 16:944–945. 10.1093/bioinformatics/16.10.944 11120685

[7] Punta M Coggill PC Eberhardt RY Mistry J Tate J Boursnell C Pang N Forslund K Ceric G Clements J Heger A Holm L Sonnhammer EL Eddy SR Bateman A Finn RD 2012 The Pfam protein families database. Nucleic Acids Res. 40:D290–D301. 10.1093/nar/gkr1065 22127870 PMC3245129

